# Root Circumnutation Reduces Mechanical Resistance to Soil Penetration

**DOI:** 10.1111/pce.15219

**Published:** 2024-10-27

**Authors:** Frederic Leuther, Daniel Iseskog, Thomas Keller, Mats Larsbo, Bipin K. Pandey, Tino Colombi

**Affiliations:** ^1^ Chair of Soil Physics University of Bayreuth Bayreuth Germany; ^2^ Department of Soil and Environment Swedish University of Agricultural Sciences (SLU) Uppsala Sweden; ^3^ Department of Agroecology and Environment Agroscope Zürich Switzerland; ^4^ School of Biosciences University of Nottingham Nottingham UK

**Keywords:** cavity expansion, plant movements, root analogues, soil biomechanics, soil structure, X‐ray computed tomography

## Abstract

Root circumnutation, the helical movement of growing root tips, is a widely observed behaviour of plants. However, our mechanistic understanding of the impacts of root circumnutation on root growth and soil exploration is limited. Here, we deployed a unique combination of penetrometer measurements, X‐ray computed tomography and time‐lapse imaging, and cavity expansion modelling to unveil the effects of root circumnutation on the mechanical resistance to soil penetration. To simulate differences in circumnutation amplitude and frequency occurring among plant species, genotypes and environmental conditions, we inserted cone penetrometers with varying bending stiffness into soil samples that were subjected to orbital movement at different velocities. We show that greater circumnutation intensity, determined by a greater circumnutation frequency in conjunction with a larger circumnutation amplitude, decreased the mechanical resistance to soil penetration. Cavity expansion theory and X‐ray computed tomography provided evidence that increased circumnutation intensity reduces friction at the cone‐soil interface, indicating a link between root circumnutation and the ability of plants to overcome mechanical constraints to root growth. We conclude that circumnutation is a key component of root foraging behaviour and propose that genotypic differences in circumnutation intensity can be leveraged to adapt crops to soils with greater mechanical resistance.

## Summary statement

We used customised penetrometers to mimic circumnutation behaviour of growing roots in soil. X‐ray tomography imaging and cavity expansion modelling provided evidence that circumnutation reduces friction at the root–soil interface, resulting in decreased mechanical resistance to soil penetration.

## 1 Introduction

Most terrestrial plants acquire water and nutrients from soil. To gain access to these vital, yet heterogeneously distributed resources (Walter, Silk, and Schurr 2009; Jin et al. 2017), plants must overcome the mechanical resistance exerted by the soil on the tip of growing roots (Jin et al. 2013; Lynch et al. 2022). Low resource accessibility due to high mechanical resistance, which typically occurs in compacted or dry soils, is a major limiting factor to plant growth and global crop productivity (Valentine et al. 2012; Colombi et al. 2018; Keller et al. 2019; Lynch et al. 2022). The already prevalent problem of soil compaction will likely aggravate due to agricultural intensification involving heavy machinery (Schjønning et al. 2015; Keller and Or 2022) and climate change will lead to more frequent and severe dry spells (IPCC 2022). Hence, identifying root traits and understanding underlying mechanisms that support root growth in hard soil is crucial to adapt crop production to denser and drier soils and to ensure future crop productivity (Colombi and Keller 2019; Lynch et al. 2022; Bello‐Bello et al. 2022).

Greater soil mechanical resistance leads to a shortening of root cortex cells (Croser, Bengough, and Pritchard 2000), which has been linked to increased stiffness of the root elongation zone (Liu et al. 2022). In turn, stiffening allows roots to exert greater penetration force, thereby facilitating root growth in hard soil (Clark et al. 2008; Schneider et al. 2021). Similarly, multiseriate cortical sclerenchyma, which are densely packed lignified cells in the outer root cortex, support the penetration of hard soil (Chimungu, Loades, and Lynch 2015; Schneider et al. 2021). Root growth in hard soil is also facilitated by traits that reduce the force needed to penetrate soil (Bengough et al. 2011; Colombi and Keller 2019). Root penetration force consists of two distinct components, namely the force needed to expand a cavity in soil and the force needed to overcome interfacial friction at the root‐soil interface (Greacen, Farrell, and Cockroft 1968; Bengough et al. 1991; Ruiz et al. 2016). A sharper root tip opening angle and the resulting shift from spherical to cylindrical soil deformation reduces the force needed for cavity expansion (Greacen, Farrell, and Cockroft 1968; Vollsnes, Futsaether, and Bengough 2010; Colombi et al. 2017; Keyes et al. 2017). Furthermore, greater mechanical resistance increases the detachment rate of root cap cells into the rhizosphere (Iijima, Griffiths, and Bengough 2000, 2003), which acts as a lubricant and thereby decreases friction at the root‐soil interface (Bengough and McKenzie 1997; McKenzie et al. 2013).

Root circumnutation, the helical movement of growing root tips, is key to the ability of root tips to respond to touch stimuli (Migliaccio, Tassone, and Fortunati 2013; Loshchilov et al. 2021) and to avoid mechanical obstacles, which facilitates plant establishment on rocky soil (Taylor et al. 2021). The intensity of root circumnutation is determined by the amplitude and the frequency of the helical movement and depends on genetic as well as environmental factors. In rice, genotypic differences in circumnutation amplitude (Taylor et al. 2021) and frequency (Inoue et al. 1999) have been found and increasing circumnutation amplitude in response to greater mechanical impedance has been observed in lentil (Martins et al. 2020). Furthermore, stiffening of the root growth zone through cell shortening (Croser, Bengough, and Pritchard 2000; Liu et al. 2022) allows roots to exert greater radial force on the soil, which may increase circumnutation amplitude.

Studies with root‐inspired robots penetrating sawdust (Del Dottore et al. 2016) or soil with a very low bulk density ($\rho_{b}$ < 0.5 g cm^−3^; Del Dottore et al. 2018) showed that mechanical resistance decreases with increasing circumnutation frequency. It was hypothesised that crack formation due to circumnutation underlies this reduction in mechanical resistance (Del Dottore et al. 2018). Moreover, circumnutation allows roots to push particles aside (Vollsnes, Futsaether, and Bengough 2010), which can lead to more cylindrical soil deformation and therefore lower cavity expansion forces (Greacen, Farrell, and Cockroft 1968; Ruiz et al. 2016). Hence, the potential of root circumnutation to reduce mechanical resistance to soil penetration has been indicated. However, these studies have been performed with very loosely packed soft substrate (Del Dottore et al. 2016, 2018) or granular material (Vollsnes, Futsaether, and Bengough 2010; Tonazzini et al. 2012), which hampers our mechanistic understanding of the role of circumnutation for root growth under mechanical conditions resembling field soil. Such insights are indispensable to identify root traits that support root growth and thus crop productivity on hard soils.

Here, we used customised cone penetrometers as root analogues that mimic the circumnutation behaviour and biomechanical properties of growing roots to elucidate hitherto poorly understood effects of root circumnutation on mechanical resistance. Experiments were conducted with remoulded field soil samples at 1.4 g cm^−3^ bulk density and 0.22 g g^−1^ water content, representing typical conditions in arable fields. Soil samples were subjected to orbital movement at different velocities to simulate different circumnutation frequencies and penetrometer probes with varying bending stiffness were used to achieve different circumnutation amplitudes. This combination of circumnutation frequencies and amplitudes allowed testing effects of circumnutation on mechanical resistance to soil penetration across a range of circumnutation intensities occurring in plant roots. We combined X‐ray computed tomography imaging and time‐lapse photography with a cavity expansion model to gain insights into the mechanisms underlying the effects of root circumnutation on the mechanical resistance to soil penetration.

## 2 Material and Methods

### 2.1 Soil Properties and Sample Preparation

We used topsoil (0–20 cm) from an arable field outside Uppsala, Sweden (59.83° N; 17.71° E) with a silt loam texture (United States Department of Agriculture 2023), an organic matter content of 41 g kg^−1^ (Supporting Information S1: Table S1), and a particle density of 2.56 g cm^−3^. The soil was passed through a 2 mm sieve, air‐dried, and rewetted to 0.20 g g^−1^ water content for sample preparation. We packed the soil in 10 mm layers into steel cylinders (diameter/height: 72/50 mm) to a height of 40 mm and a bulk density of 1.4 g cm^−3^, which is within the typical bulk density range for arable soil (Panagos et al. 2024). To ensure homogenous packing, the surface of every layer was slightly abraded before adding the next layer of soil. The bottom of the remoulded soil samples was covered with fabric mesh. Samples were slowly saturated from below for 3 days and then drained to −300 hPa matric potential, corresponding to a water content of 0.22 g g^−1^ (Supporting Information S1: Table S1). Samples were wrapped airtight and stored at 4°C until further processing.

### 2.2 Design of Penetrometer Probes and Orbital Motion Apparatus

**Figure 1:**
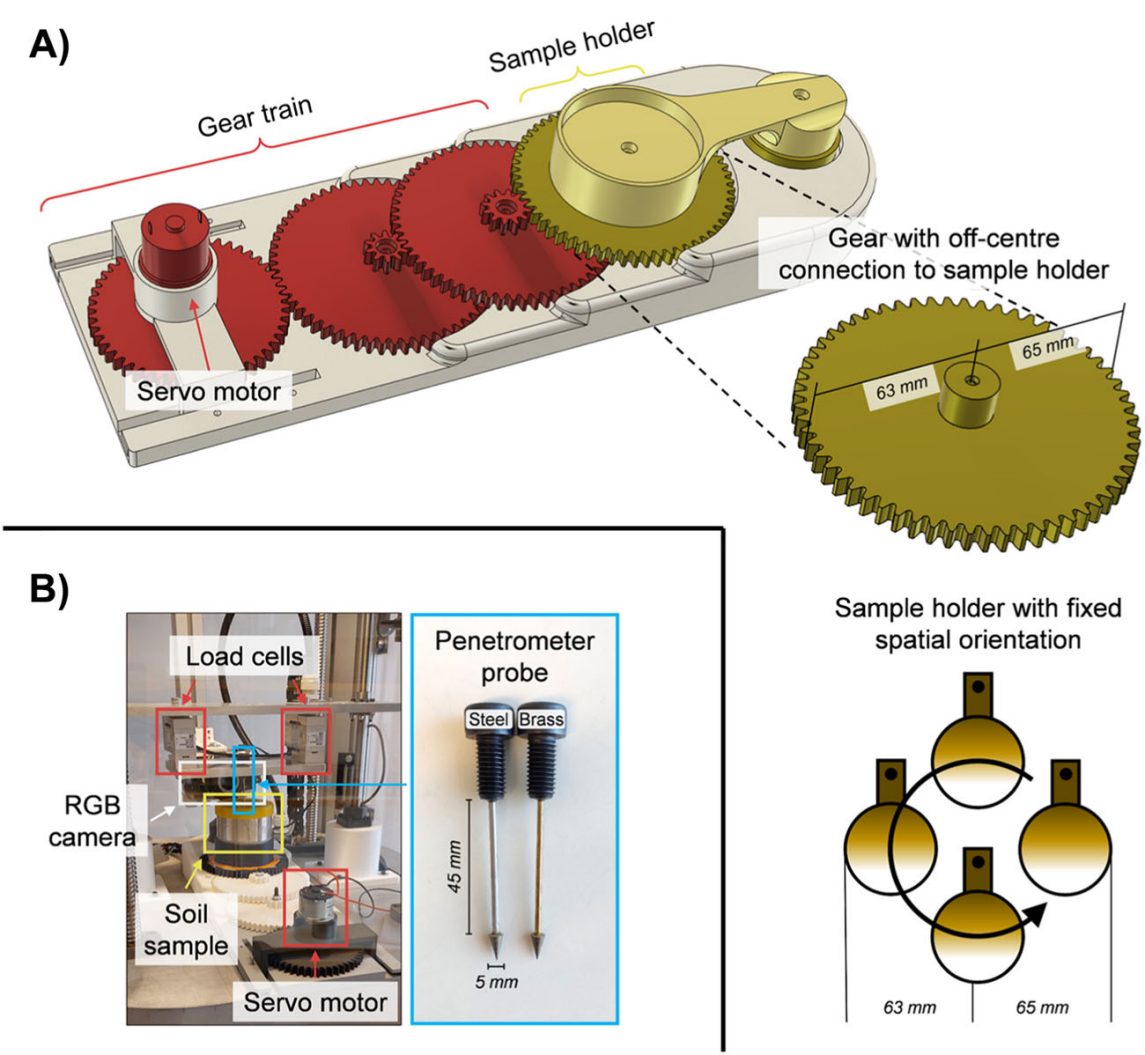
Illustration of the experimental set‐up. (A) Orbital motion apparatus including gear train allowing for 1–10 oscillations h^−1^ (note: shown configuration allowed for 5–10 oscillations h^−1^). Orbital motion was achieved by a 1 mm off‐centre connection between the last cogwheel and the sample holder. Schematic depicts the fixed spatial orientation of the sample during orbital motion. (B) Complete set‐up with orbital motion apparatus, soil sample, penetrometer probes and load cells to measure mechanical resistance, mirrorless RGB camera to quantify probe deflection, and close‐up view of penetrometer probes with steel or brass shaft.

We used customised penetrometer probes as root analogues. These probes consisted of a cone connected to a recessed shaft, representing the root tip and the root elongation zone, respectively. With a cone base radius of 2.5 mm and a shaft length of 45 mm (Figure 1B), the probes had a similar radius‐to‐length ratio as growth zones of maize (Quiros et al. 2022), wheat (Colombi et al. 2019, 2023), and poplar (Bizet, Hummel, and Bogeat‐Triboulot 2015) roots. The semi‐opening angle of the penetrometer cone was 15°, which resembled root tips of wheat (Colombi et al. 2017) and maize (Iijima, Barlow, and Bengough 2003). The cone was made of stainless steel, whereas either a 2.38 mm diameter steel rod or a 2 mm diameter brass rod (K&S Precision Metals, Chicago, IL, United States) was used for the shaft. The difference between shaft and cone base diameter (2 or 2.38 mm vs. 5 mm) ensured that no interfacial friction between the shaft and the soil occurred. We measured the force required to bend the 45 mm long penetrometer shaft with cantilever bending tests using two 50 N load cells (S2M/50 N, HBM GmbH, Darmstadt, Germany; Accuracy: 0.02%). The bending stiffness of the shaft (k) was then given by the slope of the bending force as a function of the deflection distance. Measurements of root stiffness (cantilever bending tests) and root diameter reported in the literature (Dexter and Hewitt 1978) were used to compare bending behaviour between the penetrometer probes used here and plant roots.

To mimic root circumnutation, we manufactured an orbital motion apparatus using 3D printing. We used a servo motor (6 rpm Gear Motor, Servocity, Winfield, KS, United States) connected to a gear train to control the velocity of the orbital motion. The last cogwheel of the gear train was connected to a sample holder, which had a fixed orientation to prevent the soil sample from revolving around its own axis. The connection between the last cogwheel and the sample holder was placed 1 mm off the centre of the cogwheel to induce orbital motion of the soil sample. All cogwheels were equipped with ball bearings to ensure smooth rotation. The configuration of the gear train enabled orbital motion in the range of 1–10 oscillations per hour (Figure 1A). The sample holder and the mechanism to induce orbital motion were made from carbon fibre re‐enforced nylon (Onyx, Markforged, Watertown, MA, United States of America), while the remaining parts were made from Polylactide (PLA) plastic (X‐PLA, Add North 3D AB, Ölsremma, Sweden).

### 2.3 Soil Penetrometer Tests

Soil samples were placed into the sample holder and covered with a plastic lid to avoid evaporation. The penetrometer probe was inserted into the sample through a 6 mm diameter hole in the centre of the lid. Before starting mechanical resistance measurements, the cone was fully inserted into the soil sample. Penetrometer probes were then inserted 1 cm deep into the sample at a penetration speed of 1 cm h^−1^. Axial penetration force ($F_{z}$) was recorded every second with two 50 N load cells (S2M/50 N, HBM GmbH, Darmstadt, Germany; Accuracy: 0.02%) that were connected via an aluminium plate to the penetrometer probe (Figure 1B). Dividing penetration force by the cone base area yielded mechanical resistance. Soil samples remained either stationary during penetration tests or were subjected to orbital motion at 1, 5 and 10 oscillations h^−1^. Given the penetration speed of 1 cm h^−1^, orbital motion at these three velocities simulated circumnutation frequencies (f) of 1, 5 and 10 cm^−1^, which is in the range of root circumnutation frequencies reported for rice (Taylor et al. 2021), wheat (Colombi et al. 2023), pea (Kim et al. 2016), maize (Del Dottore et al. 2016) and lentils (Martins et al. 2020). All shaft material‐circumnutation frequency combinations were replicated five times (*n* = 5). After measurements, samples were wrapped airtight and stored at 4°C until further processing.

### 2.4 Quantification of Probe Deflection

We fixed a 24 megapixel mirror‐less camera (Canon EOS M6, Canon, Tokyo, Japan) perpendicular to the penetrometer probe and the soil sample (Figure 1B). The camera was equipped with a macro lens (EF‐M 28 mm f1/3 IS STM, Canon, Tokyo, Japan), resulting in 10.8 µm pixel edge length. The aperture value, exposure time and film speed were set manually (Supporting Information S1: Table S2) and the camera shutter was controlled with an Arduino microcontroller (Arduino Mega 2560, Arduino AG, Somerville, MA, United States). Pictures were taken every minute during penetrometer tests. ImageJ (version 1.53e; National Institute of Health, Bethesda, MD, United States) was used to measure the projected probe deflection angle. We manually measured the angle 20 mm from the upper end of the probe shaft to the vertical in every picture. The difference between the maximum and minimum projected deflection angle during one sample oscillation was used to calculate the average horizontal deflection of the cone ($\delta_{c}$) as:

(1) $$\delta_{c}=\sum_{i}^{R}\sin\left(\frac{{∆\alpha }_{i}}{2}\right)l_{s}\frac{1}{R},$$

where $l_{s}$ denotes the length of the shaft of the penetrometer probe ($l_{s}$ = 45 mm), R denotes the total number of oscillations during penetrometer tests (R = 1, 5 or 10), and ${∆\alpha }_{i}$ denotes the difference between the maximum and minimum projected deflection angle during the $i^{\mathrm{th}}$ oscillation. The circumnutation amplitude was given by the difference between the orbital movement radius and the horizontal cone deflection (i.e. $1-\delta_{c}$). Since our set‐up measured penetration force in vertical direction ($F_{z}$; Figure 1B), horizontal deflection of the probe leads to an underestimation of the actual axial penetration force ($F_{a}$). We calculated $F_{a}$ as a function of $F_{z}$ and ${∆\alpha }_{i}$ as follows:

(2) $$F_{a}={F_{z}\;\left(\sum_{i}^{R}\cos\left(\frac{∆\alpha_{i}}{2}\right)\frac{1}{R}\right)}^{-1}.$$

The relative error ($e_{\mathrm{rel}}$) of force measurements caused by probe deflection is then:

(3) $$e_{\mathrm{rel}}=\frac{F_{a}{-F}_{z}}{F_{z}}\times 100\%.$$

### 2.5 X‐Ray Computed Tomography to Quantify Soil Structure

Subsamples for X‐ray computed tomography scanning were taken with aluminium cylinders (inner diameter/wall thickness: 18/1 mm) that were sharpened at one end. The aluminium cylinders were inserted at a constant speed (4 mm s^−1^) into the centre of the 72 mm diameter soil sample. Lateral movement of the aluminium cylinder and the soil sample was constrained during sampling to prevent structural damage. In addition to samples subjected to penetrometer tests, we took subsamples from five 72 mm diameter samples that had not been subjected to penetrometer tests. The uppermost 24 mm of the subsamples were scanned in an industrial X‐ray scanner (GE Phoenix v|tome|x m; GE Inspection Technologies, Lewistown, PA, USA) at 12 µm resolution. Image stacks of reconstructed scans were exported as 16‐bit.tiff files. Further image acquisition and reconstruction parameters are provided in Supporting Information S1: Table S3. After scanning, subsamples and the remaining bulk soil were dried for 72 h at 105°C to determine gravimetric water content and dry bulk density.

X‐ray scans were processed and analysed in FIJI ImageJ (Schindelin et al. 2012). Specific image processing parameters are provided in Supporting Information S1: Table S4. We normalised the grey value distribution of the images between the aluminium cylinder wall and the air‐filled pore space using the SoilJ plugin (Koestel 2018). Images were then converted to 8‐bit greyscale and filtered with a nonlocal means and unsharp mask filter (Buades, Coll, and Morel 2011; Schlüter et al. 2014). Grey value normalisation enhanced contrast in the images, which enabled segmentation into binary images of pores and soil matrix with one global threshold for all samples (Koestel et al. 2021). The outermost 0.6 mm (i.e., 50 voxels) of the soil sample were excluded from further analyses to avoid potential sampling artifacts.

The volume of pore clusters was used to isolate the cone imprint from all other visible macropores ( ≥ 12 µm diameter). To do so, the binary image was scaled by factor 0.5 and filtered with a median3D filter. This removed all pores with a diameter smaller than 120 µm, representing most of the soil pore network (Supporting Information S1: Figure S1A–C). A watershed algorithm followed by two iterations of voxel erosion and dilation spatially separated the imprint of the cone from the remaining soil pore space while maintaining the total imprint volume. The different clusters were labelled by connected component labelling (Legland, Arganda‐Carreras, and Andrey 2016) and the volume of every individual cluster was determined. Being the largest pore cluster by at least one order of magnitude, the cone imprint could be readily identified as the largest individual cluster. The image was rescaled to the original size, followed by a second iteration of watershed segmentation, erosion and dilation to remove remaining pore clusters connected to the cone imprint (Supporting Information S1: Figure S1D,E). Manual quality checks were performed for every sample. Subtracting the isolated cone imprint from the rest of the segmented pore system allowed obtaining structural information of the soil around and below the cone imprint. Pore network indicators were calculated for the original image resolution, i.e., for pores ≥ 12 µm in diameter.

Soil structural properties were quantified separately for the volume below the tip of the cone imprint (1000 mm^3^) and the volume around cone imprint (2000 mm^3^) to distinguish between axial and radial impacts of penetrometer tests. Standard pore characteristics including visible porosity and mean pore diameter were used to describe the soil pore space of the two volumes following the protocols presented by Weller et al. (2022). To obtain spatially explicit information on soil deformation patterns, visible porosity was quantified as a function of the distance from the cone imprint. To this end, the cone imprint was dilated in five voxel iterations (i.e., 60 µm) and visible porosity was quantified for every iteration, yielding visible porosity profiles at 60 µm increments. We used these radial porosity profiles to calculate the relative difference in soil compactness (∆c) as a metric for local soil deformation patterns around the cone imprint:

(4) $$∆c=1-\frac{{\varepsilon^{\prime }}_{\mathrm{vis}}}{\varepsilon_{\mathrm{vis}}},$$

where $\varepsilon_{\mathrm{vis}}$ is the visible porosity before penetrometer tests, given by the average visible porosity of all five samples not subjected to penetrometer tests, and ${\varepsilon^{\prime }}_{\mathrm{vis}}$ is the visible porosity after penetrometer tests. Moreover, we used the change in visible porosity caused by penetrometer tests (${∆\varepsilon }_{\mathrm{vis}}$) to estimate local effects of penetrometer tests on total porosity ($\varepsilon^{\prime }$) and soil bulk density (${\rho^{\prime }}_{b}$):

(5) $$\varepsilon^{\prime }=\varepsilon -{∆\varepsilon }_{\mathrm{vis}}=\left(1-\frac{\rho_{b}}{\rho_{p}}\right)-{(\varepsilon }_{\mathrm{vis}}-{\varepsilon^{\prime }}_{\mathrm{vis}}),$$

(6) $${\rho^{\prime }}_{b}=\left(1-\varepsilon \prime \right)\rho_{p},$$

where ε denotes total porosity before penetrometer tests (ε = 0.45 m^3^ m^−3^), $\rho_{b}$ is the bulk density before penetrometer tests ($\rho_{b}$ = 1.4 g cm^−3^), and $\rho_{p}$ is the particle density ($\rho_{p}$ = 2.56 g cm^−3^). Since $\varepsilon^{\prime }$ and ${\rho^{\prime }}_{b}$ were estimated from ${∆\varepsilon }_{\mathrm{vis}}$, changes of non‐visible porosity ( < 12 µm diameter) were neglected. Hence, $\varepsilon^{\prime }$ and ${\rho^{\prime }}_{b}$ may have slightly differed from actual porosity and bulk density values.

### 2.6 Modelling Penetration Force Components

Axial penetration force measured with a cone penetrometer is the sum of the axial contribution from cavity expansion and frictional forces (Greacen, Farrell, and Cockroft 1968; Bengough et al. 1991; Ruiz et al. 2016). For samples that remain stationary during penetrometer tests, the radial force exerted by the cone ($F_{r,s}$) is (Ruiz et al. 2016):

(7) $$F_{r,s}=\pi {r_{c}}^{2}\cot\beta_{c}\sigma_{r,s},$$

where $r_{c}$ and $\beta_{c}$ are the cone base radius and semi‐opening angle, respectively, and $\sigma_{r,s}$ is the radial stress exerted on the cone during penetrometer tests with stationary samples. The frictionless axial cavity expansion force acting on the cone surface for stationary samples ($F_{c,s}$) is then (Ruiz et al. 2016):

(8) $$F_{c,s}=F_{r,s}\tan\beta_{c}=\pi {r_{c}}^{2}\sigma_{r,s}.$$

The sum of $F_{c,s}$ and the frictional force in axial direction of stationary samples ($F_{f,s}$) then yields the total axial force measured with a penetrometer ($F_{z}$) (Ruiz et al. 2016):

(9) $$F_{z}=F_{c,s}+F_{f,s}=F_{c,s}\left[1+\mu \cot\beta_{c}\right]=\pi {r_{c}}^{2}\sigma_{r,s}\left[1+\mu \cot\beta_{c}\right],$$

where μ is the metal‐soil friction coefficient. Based on mean $F_{z}$ values (*n* = 5) obtained from penetrometer tests using probes with steel or brass shafts inserted into stationary samples ($\overset{®}{F_{z}}$), $\sigma_{r,s}$ was calculated as:

(10) $$\sigma_{r,s}=\frac{\bar{F_{z}}}{\pi {r_{c}}^{2}\left[1+\mu \cot\beta_{c}\right]}.$$

**Figure 2:**
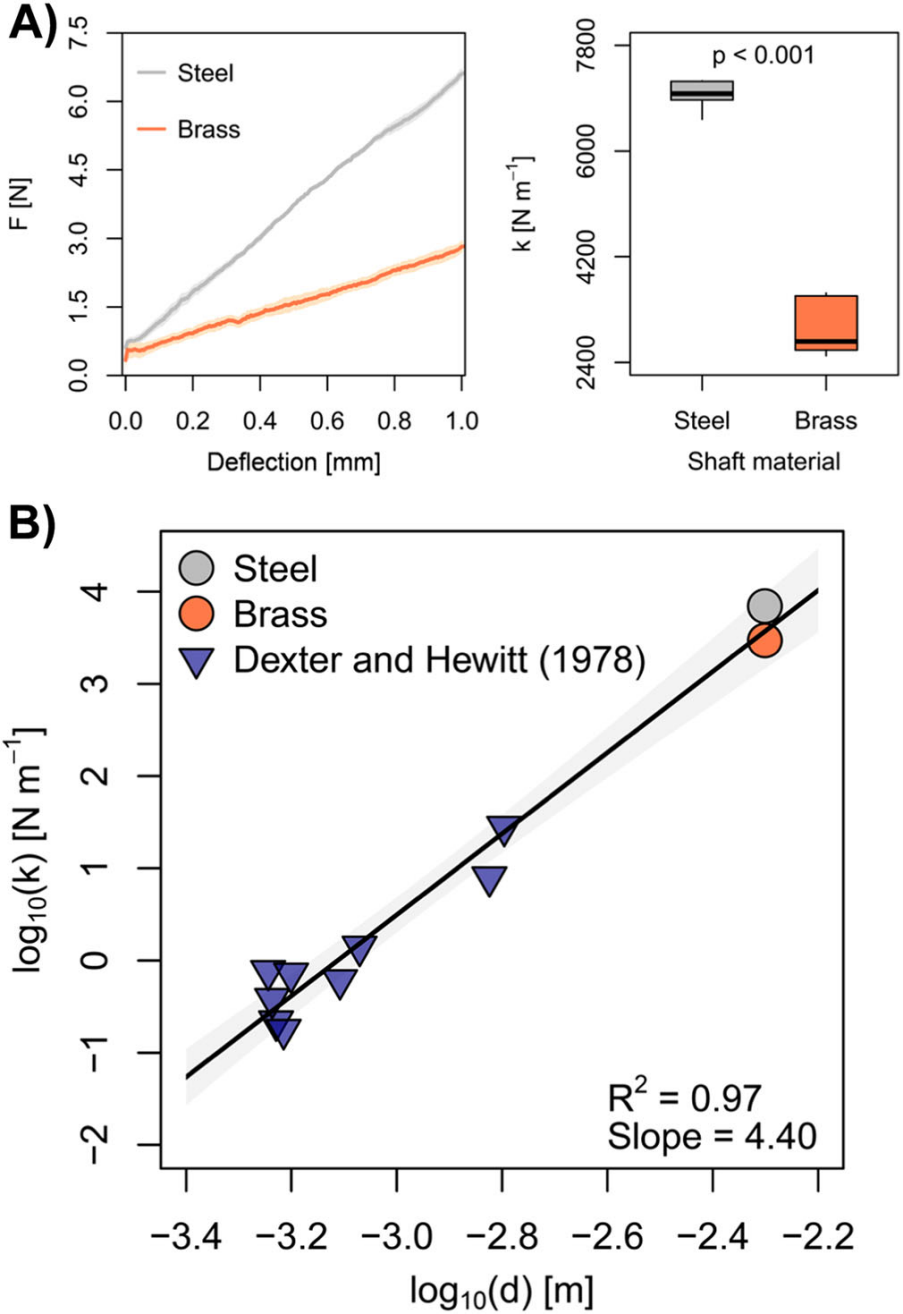
Bending properties of penetrometer probes and plant roots. (A) Bending force (F) as a function of probe deflection at the cone base and bending stiffness (k) of penetrometer probes with a steel (i.e. stiff) or brass (i.e., flexible) shaft. Shading denotes standard error and p‐value was obtained from Student's t‐test (*n* = 6). (B) Scaling of diameter (d) with bending stiffness (k) for roots reported in literature (triangles) and penetrometer probes used in the current study (circles). Root data was taken from Dexter and Hewitt (1978). Solid line and shading depict regression line and 95% confidence interval of linear regressions, respectively, and *R* ^2^ denotes the coefficient of determination. [Color figure can be viewed at wileyonlinelibrary.com]

Orbital motion of the sample results in an additional unidirectional radial force ($F_{\mathrm{ro}}$) acting on the probe during penetration. This results in an asymmetric distribution of the radial forces around the circumferential axis of the cone. $F_{\mathrm{ro}}$ is given by the horizontal deflection of the penetrometer cone during penetrometer tests ($\delta_{c}$; Equation 1) and the bending stiffness of the probe shaft (k; Figure 2A):

(11) $$F_{\mathrm{ro}}=\delta_{c}k.$$

The radial force at any position around the circumferential axis of the penetrometer (${F^{*}}_{r}$) is then:

(12) $${F^{*}}_{r}\left(\gamma \right)=F_{r,s}+\cos\gamma F_{\mathrm{ro}};F_{r,s}\geq F_{\mathrm{ro}},$$

where γ denotes the angle between ${F^{*}}_{r}$ and $F_{\mathrm{ro}}$. Assuming similar net cavity size and soil compression patterns around the cone between stationary samples and samples subjected to orbital movement, radial ($F_{r,m}$) and axial cavity expansion force ($F_{c,m}$) for samples subjected to orbital movement are given by:

(13) $$F_{r,m}=F_{r,s},$$

(14) $$F_{c,m}=F_{c,s},$$

Similar as rotating a penetrometer around its own axis, orbital movement of the sample leads to a frictional force vector that is more perpendicular to the probe axis, which decreases frictional force (Bengough et al. 1991, 1997; McKenzie et al. 2013). This similarity between a rotating penetrometer and orbital movement of the sample suggests that orbital movement alters frictional forces at the cone‐soil interface. Following Equations (9) and (14), frictional force for samples subjected to orbital motion ($F_{f,m}$) is given by:

(15) $$F_{f,m}=F_{z}-F_{c,s}.$$

Since interfacial friction coefficients differ substantially between metal cones and roots (Bengough and McKenzie 1997; Bengough, Mullins, and Wilson 1997; McKenzie et al. 2013), we calculated total axial penetration force ($F_{z}^{\prime }$) as a function of the friction coefficient (μ′) following Colombi et al. (2017):

(16) $$F_{z}^{\prime }\left(\mu \prime \right)=F_{z}\frac{\left[1+\mu \prime \cot\beta_{c}\right]}{\left[1+\mu \cot\beta_{c}\right]}.$$

$F_{f}^{\prime }$ can be readily calculated as a function of μ′:

(17) $$F_{f}^{\prime }\left(\mu \prime \right)=F_{z}^{\prime }\left(\mu \prime \right)-F_{c,s},$$

Given the dimensions of the penetrometer cone used here, $r_{c}$ was 2.5 mm and $\beta_{c}$ was 15°; μ was set to 0.5 and μ′ ranged from 0.1 to 0.5, representing typical friction coefficients for boundary lubricants (Hutchings 1992) and metal–soil interfaces (Bengough, Mullins, and Wilson 1997).

### 2.7 Data Analysis and Statistics

We quantified the degree of asymmetry in the distribution of the radial force around the circumferential axis of the penetrometer cone with an asymmetry index ($I_{\mathrm{asym}}$):

(18) $$I_{\mathrm{asym}}=\frac{{F^{*}}_{r}\left(0\right)-{F^{*}}_{r}\left(\pi \right)}{2F_{r,s}}.$$

Due to the boundary conditions set in Equation 12, $I_{\mathrm{asym}}$ is between 0 and 1 with increasing $I_{\mathrm{asym}}$ indicating greater asymmetry. To quantify circumnutation intensity, we merged $I_{\mathrm{asym}}$ with the circumnutation frequency into a single circumnutation intensity index (CI):

(19) $$\mathrm{CI}=I_{\mathrm{asym}}\frac{f}{f_{\max}},$$

where f is the circumnutation frequency during the penetrometer test (f = 0, 1, 5 or 10 cm^−1^) and $f_{\max}$ is the maximum circumnutation frequency tested in the study ($f_{\max}$ = 10 cm^−1^).

R version 4.0.2 (R Core Team 2020) was used for statistical analyses. Effects of the circumnutation frequency, the shaft material of the penetrometer probes, and their interaction were analysed with the analysis of co‐variance models including the circumnutation frequency as a continuous variable and the shaft material as a categorical variable. Treatment means were compared with least significant difference (LSD) tests as implemented in the ‘agricolae’ package (de Mendiburu 2017). Regressions were evaluated with the least‐squares method from the ‘stats’ package (R Core Team 2020). Model residuals were tested for normal distribution with Shapiro–Wilk tests.

## 3 Results

### 3.1 Circumnutation Amplitude Increases With Probe Stiffness

**Figure 3:**
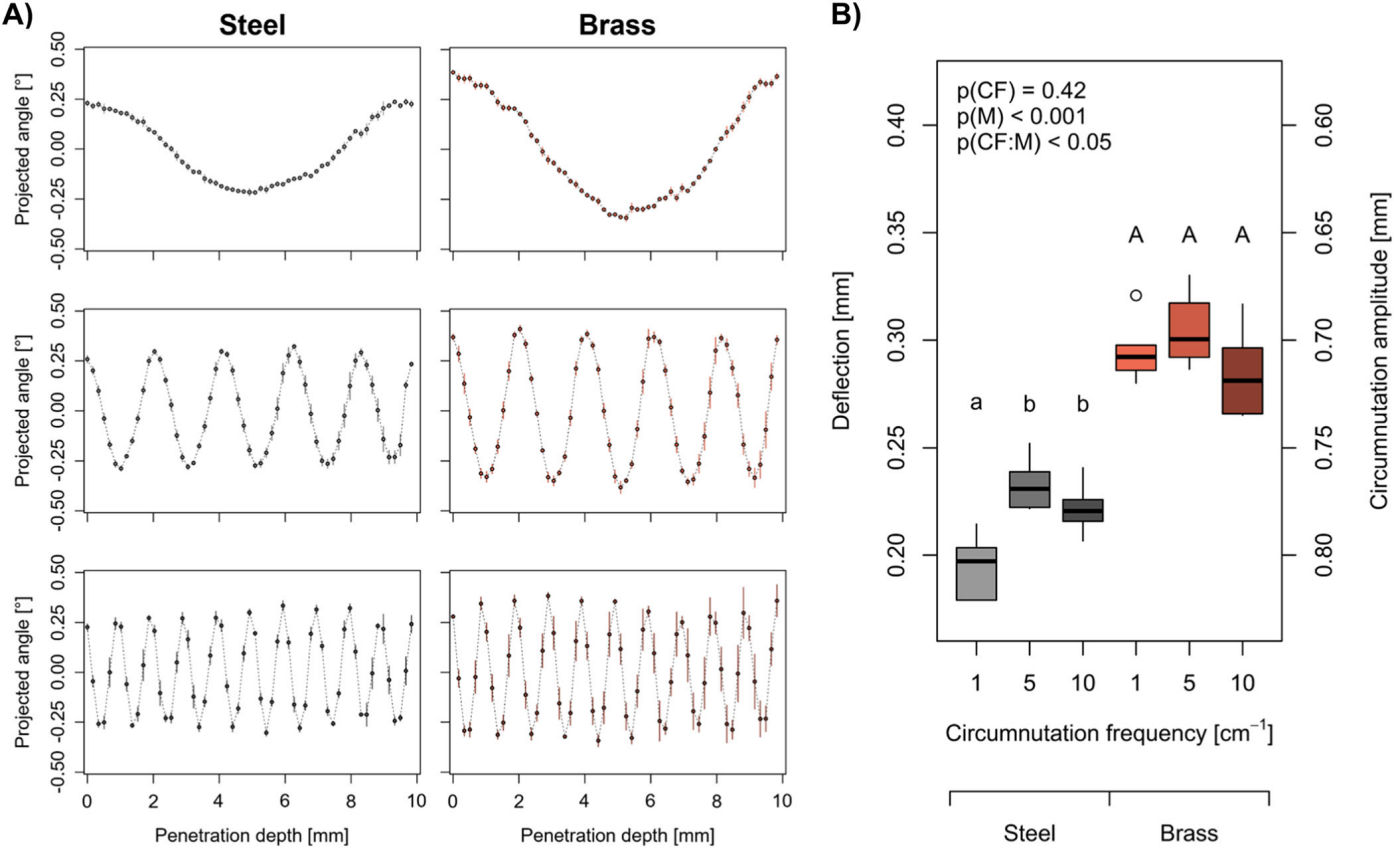
Deflection of penetrometer probes in response to circumnutation. (A) Projected deflection angle to horizontal of penetrometer probe with steel (i.e., stiff) or brass (i.e., flexible) shaft quantified from images taken perpendicular to penetrometer probe at 1‐min intervals. Panels depict deflection angles at circumnutation frequencies of (top) 1, (middle) 5 and (bottom) 10 oscillations cm^−1^ of penetration. Error bars denote standard error (*n* = 5). B) Average horizontal deflection of penetrometer cone connected to steel (i.e., stiff) or brass (i.e., flexible) shaft and corresponding circumnutation amplitude at different circumnutation frequencies. *p*‐values were obtained from analysis of co‐variance model testing effects of circumnutation frequency (CF), shaft material (M), and their interaction (CF:M) on mechanical resistance. Different letters indicate significant differences within shaft materials according to least significance difference (LSD) tests at *p* = 0.05 (*n* = 5).

The bending stiffness (k) of the steel shaft was more than double than that of the brass shaft ($k_{\mathrm{steel}}$ = 6.96 kN m^−1^, $k_{\mathrm{brass}}$ = 2.96 kN m^−1^; Figure 2A). The logarithms of the bending stiffness of the penetrometer and the cone diameter scaled linearly with the logarithms of the bending stiffness (cantilever bending tests) and the diameter of roots reported in the literature (Dexter and Hewitt 1978; slope = 4.40 ± 0.24 (standard error), *R* ^2^ = 0.97, Figure 2B). The deflection angle of the probe quantified from time‐lapse images did not change with penetration depth (Figure 3A), indicating constant horizontal deflection of the penetrometer cone across penetration depths. The horizontal deflection of the penetrometer cone and thus the circumnutation amplitude differed significantly between shaft materials (*p* < 0.001). Probes with the more flexible brass shaft had a circumnutation amplitude of around 0.70 mm, whereas the circumnutation amplitude of probes with the stiffer steel shaft was between 0.7 and 0.81 mm (Figure 3B).

### 3.2 Mechanical Resistance Deceases With Circumnutation

**Figure 4:**
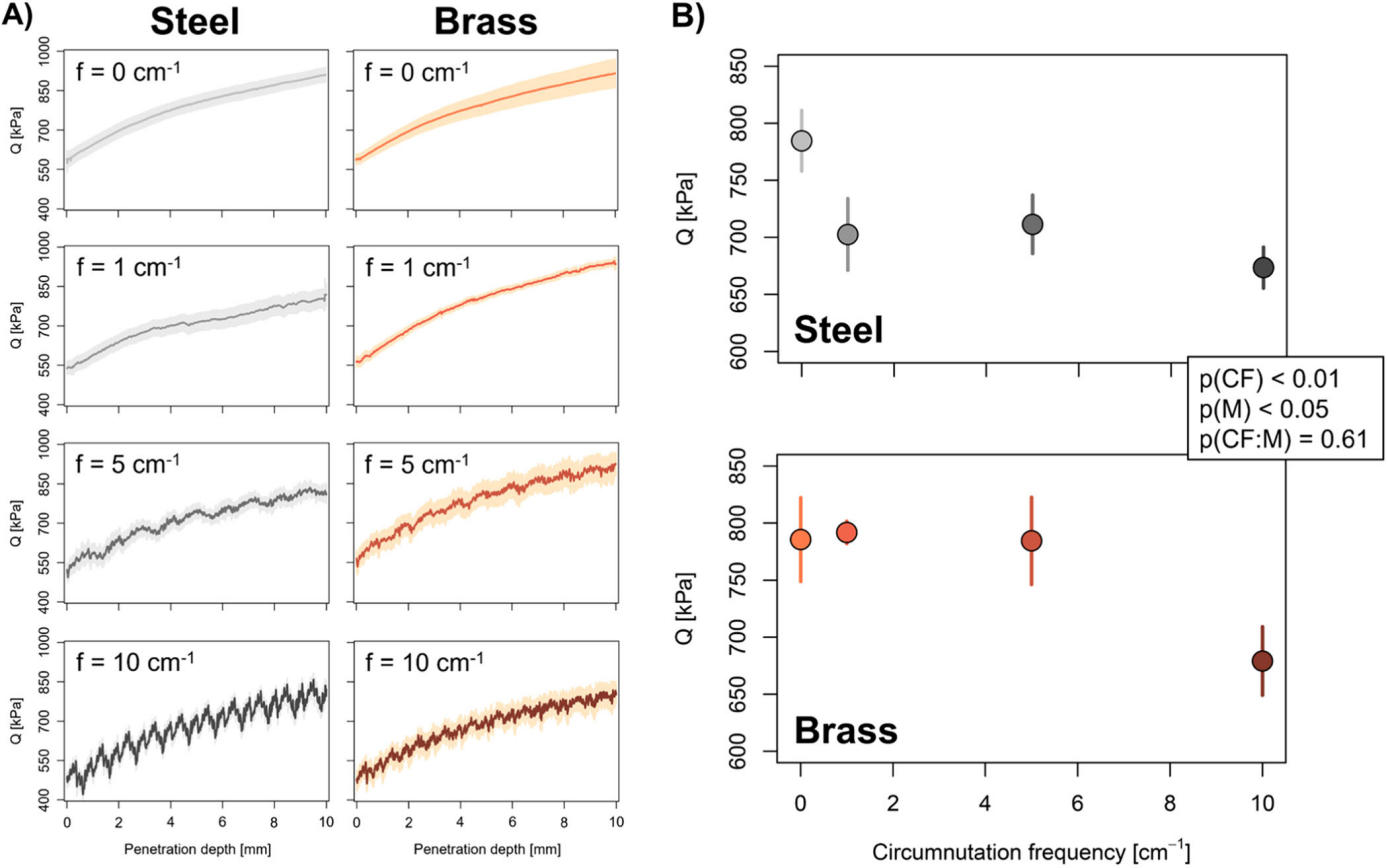
Effects of circumnutation frequency and shaft material of penetrometer probes on mechanical resistance to soil penetration. (A) Mechanical resistance (Q) as a function of penetration depth in static samples (f = 0 oscillations cm^−1^) and samples subjected to orbital motion (f = 1, 5 or 10 oscillations cm^−1^) for penetrometer probes with a steel (i.e., stiff) or brass (i.e., flexible) shaft. Solid line and shading denote mean value and standard error, respectively (*n* = 5). (B) Mean mechanical resistance (0 to 10 mm depth) as a function of circumnutation frequency for penetrometer probes with a steel (i.e., stiff) or brass (i.e., flexible) shaft. Error bars denote standard error and *p*‐values were obtained from analysis of co‐variance model testing effects of circumnutation frequency (CF), shaft material (M) and their interaction (CF:M) on mechanical resistance (*n* = 5). [Color figure can be viewed at wileyonlinelibrary.com]

We measured axial penetration force from the point the cone was fully inserted into the soil until 1 cm penetration depth (Figure 4A). The relative error of force measurements due to radial deflection of the penetrometer probe (Equations 2 and 3) was below 0.003% and thus negligible (Supporting Information S1: Table S5). Mean mechanical resistance across the entire penetration depth of 1 cm was significantly affected by the circumnutation frequency (*p* < 0.01) and the shaft material (*p* < 0.05; Figure 4B). In samples that remained stationary during penetrometer tests, mean mechanical resistance was around 0.79 MPa for both shaft materials. Differences between shaft materials occurred at circumnutation frequencies of one and five oscillations per centimetre of penetration. Mechanical resistance to soil penetration of probes with a comparatively stiff steel shaft decreased by 10% to around 0.71 MPa. By contrast, mechanical resistance of probes with a more flexible brass shaft were not affected by circumnutation frequencies of one and five oscillations per centimetre of penetration. Hence, the differences in bending stiffness and the resulting variability in circumnutation amplitude between the stiffer steel and more flexible brass shaft affected mechanical resistance at circumnutation frequencies of one and five oscillations per centimetre of penetration. Mechanical resistance decreased by around 15% to 0.67 MPa for both shaft materials at a circumnutation frequency of 10 oscillations per centimetre of penetration (Figure 4B).

### 3.3 No Effects of Circumnutation on Soil Structure

**Figure 5:**
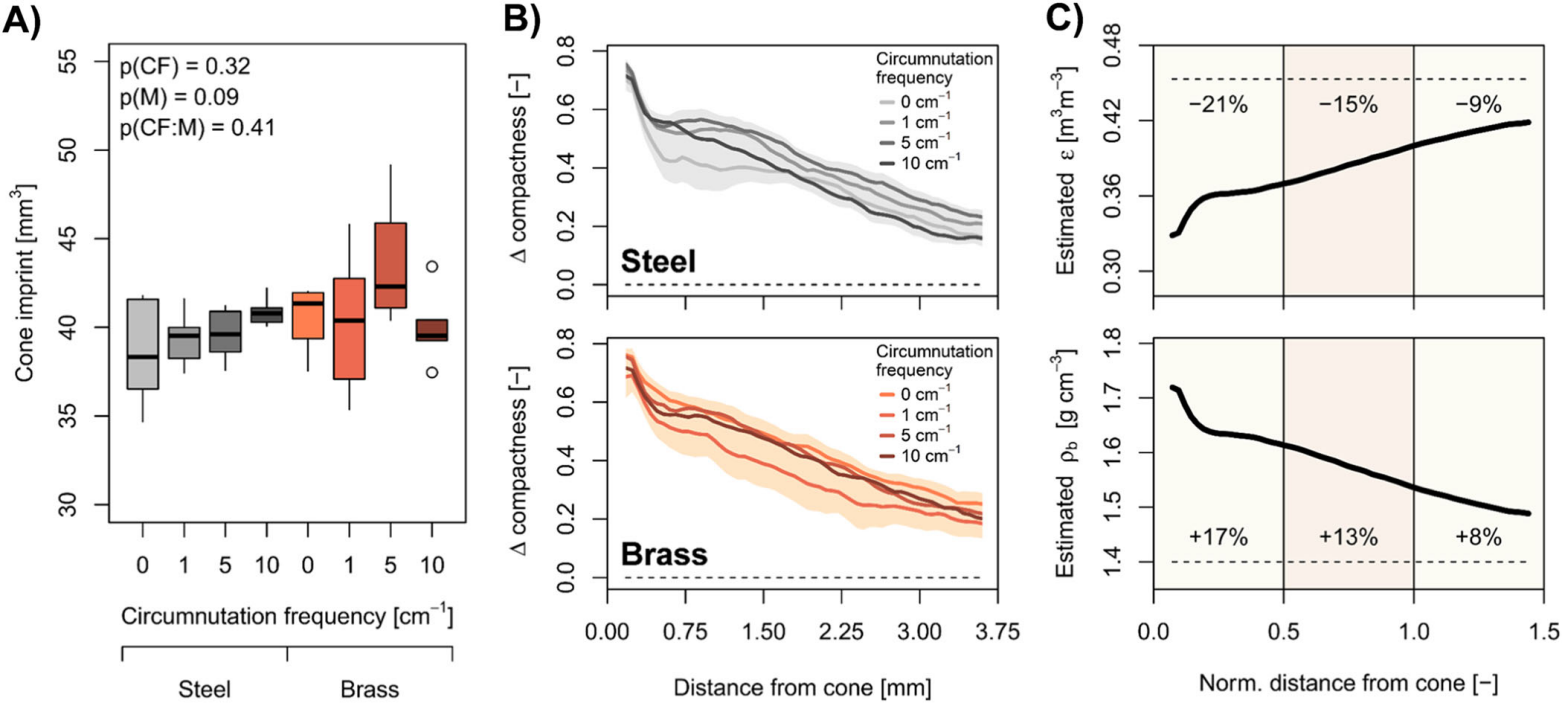
Soil structural features obtained and derived from X‐ray computed tomography scans at a resolution of 12 µm. (A) Effects of circumnutation frequency (CF), shaft material (M), and their interaction (CF:M) on the size of the cone imprint evaluated with analysis of co‐variance models (*n* = 5). (B) Relative difference in soil compactness around the cone imprint, calculated using visible porosity (Equation 4), as a function of the distance from the surface of the cone imprint. Dashed line indicates compactness of samples not subjected to penetrometer tests and shading denotes standard error (*n* = 5). (C) Estimated total porosity (ε; Equation 5) and estimated soil bulk density ($\rho_{b}$; Equation 6) calculated from visible porosity around the cone imprint as a function of the normalised distance from the surface of the cone imprint. Beige and brown segments denote 0.5 cone radius increments. Solid line denotes average value including both shaft materials and all four circumnutation frequencies and dashed line indicates ε and $\rho_{b}$ of samples not subjected to penetrometer tests. [Color figure can be viewed at wileyonlinelibrary.com]

The central 18 mm diameter subsamples used for X‐ray computed tomography had the same soil moisture and mean bulk density as the surrounding bulk soil (Supporting Information S1: Table S1). Moreover, neither soil moisture nor soil bulk density differed among circumnutation frequencies and shaft materials (coefficient of variation: < 2.4%; Supporting Information S1:Figure S2). The average size of the cone imprint was around 40 mm^3^ and no significant effects of circumnutation frequency (*p* = 0.32) or shaft material (*p* = 0.09) occurred (Figure 5A).

Soil porosity visible at 12 µm resolution and mean pore diameter in the soil volume around and below the cone imprint did not differ significantly among circumnutation frequencies (*p* ≥ 0.12) and shaft materials (*p* ≥ 0.08). Moreover, visible porosity and mean pore diameter below the cone imprint were very similar to samples not subjected to penetrometer tests, indicating at best marginal axial effects of penetrometer tests on soil structure (Supporting Information S1: Figure S3). Soil compactness as a function of the distance to the cone imprint derived from visible porosity profiles (Supporting Information S1: Figure S4, Equation 4) provided further insights into radial effects of penetration tests on soil structure. In the first 1.25 mm from the cone imprint, representing half of the cone radius, soil compactness increased by around 55% (Figure 5B). This corresponded to a decrease in estimated total soil porosity of 21% (Equation 5) and an increase in estimated soil bulk density of 17% (Equation 6; Figure 5C). The magnitude of these impacts decreased with the distance to the cone imprint, yet clear effects of penetrometer tests on soil structure occurred more than 2.5 mm away from the cone imprint (Figure 5B,C). However, soil compactness profiles did not differ among shaft materials and circumnutation frequencies (Figure 5B), indicating similar soil deformation patterns across circumnutation intensities. Hence, neither cavity size nor soil structural properties around and below the cavity formed by the penetrometer were affected by circumnutation frequency or amplitude.

### 3.4 Greater Circumnutation Intensity Reduces Frictional Force

**Figure 6:**
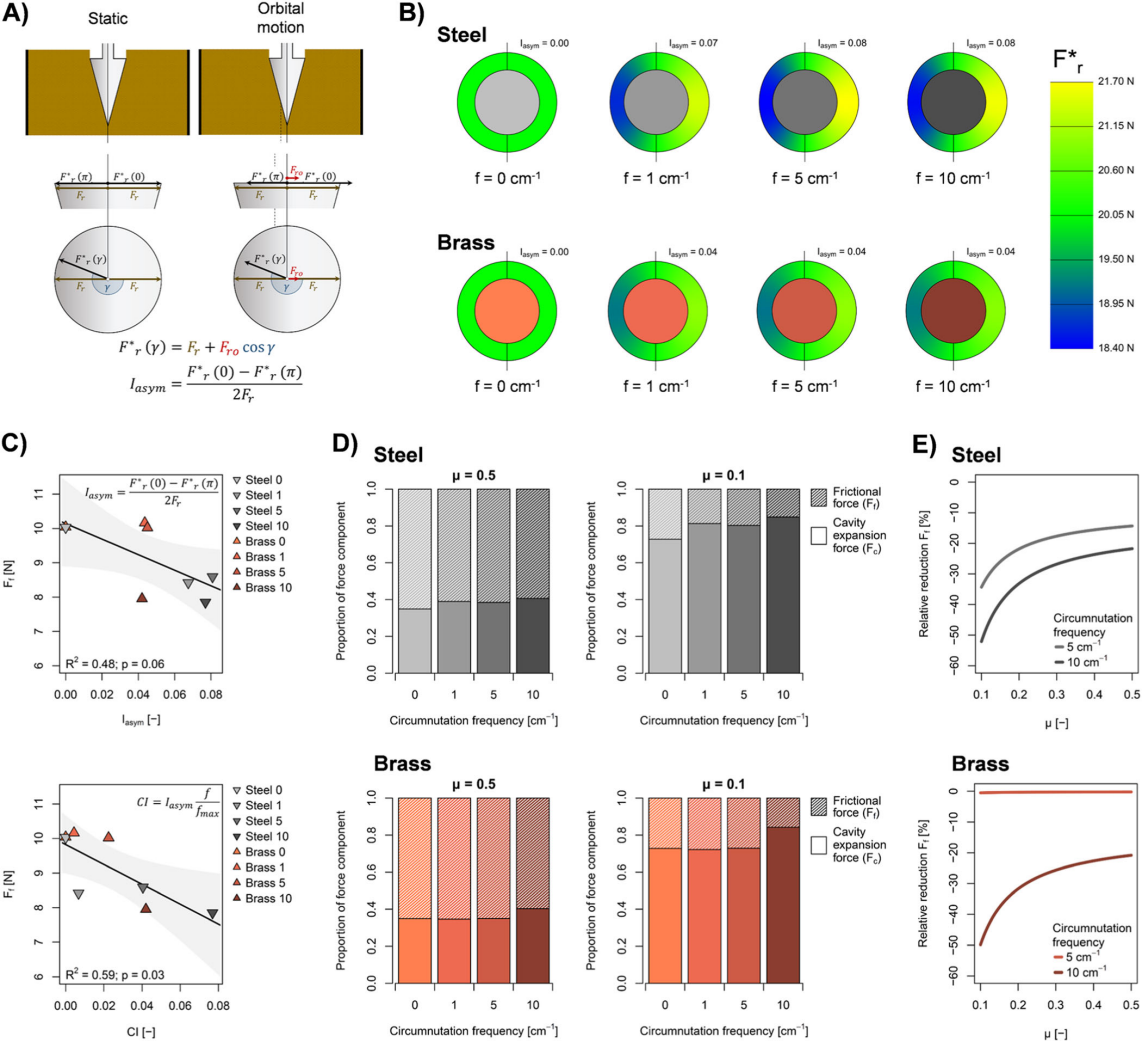
Penetration force components obtained from cavity expansion modelling. (A) Schematic representation of the effects of the unidirectional force ($F_{\mathrm{ro}}$) resulting from orbital motion of the sample on the distribution of the radial force ($F_{r}$) around the circumferential axis of the penetrometer. An asymmetry index ($I_{\mathrm{asym}}$) describes the asymmetric distribution of the radial force (${F^{*}}_{r}$) caused by $F_{\mathrm{ro}}$. (B) Distribution of ${F^{*}}_{r}$ around the circumferential axis of penetrometer probes with a steel (i.e., stiff) or brass (i.e., flexible) shaft at different circumnutation frequencies (f). (C) Frictional force ($F_{f}$) as a function of (top) $I_{\mathrm{asym}}$ and (bottom) circumnutation intensity (CI). Solid line and shading depict regression line and 95% confidence interval of linear regressions, respectively, and *R* ^2^ denotes the coefficient of determination. (D) Relative contribution of $F_{f}$ and cavity expansion force ($F_{c}$) to total axial penetration force for probes with steel (i.e., stiff) or brass (i.e. flexible) shafts at different circumnutation frequencies and for two friction coefficients (μ) typical for metal‐soil (μ = 0.5) and root‐soil (μ = 0.1) interfaces. (E) Relative reduction of $F_{f}$ resulting from circumnutation at f = 5 and 10 cm^−1^ for probes with steel (i.e., stiff) or brass (i.e., flexible) shafts as a function of μ. Data displayed in (B) to (E) were calculated from penetration force measurements (Equations (7), (8), (9), (10), (11), (12), (13), (14), (15), (16), (17), (18), (19)) and represent mean values (*n* = 5). [Color figure can be viewed at wileyonlinelibrary.com]

The cavity expansion model used in the current study showed that circumnutation resulted in an asymmetric distribution of radial cavity expansion forces around the circumferential axis of the penetrometer. The total cavity expansion force, however, remained unaffected by circumnutation (Figure 6A). Shaft stiffness (Figure 2A) and the horizontal deflection of the penetrometer probe (Figure 3B) allowed quantification of this asymmetry (Equation 18). This revealed a greater degree of asymmetry in the distribution of radial cavity expansion forces around the circumferential axis of the penetrometer for probes with the stiffer steel than the more flexible brass shaft (Figure 6B). Furthermore, our cavity expansion model indicated that circumnutation affected frictional forces at the cone‐soil interface that occurred during penetrometer tests. The circumnutation intensity index, determined by the degree of asymmetry in the distribution of the radial force around the cone and the circumnutation frequency (Equation 19), was negatively correlated with the frictional force (*R* ^2^ = 0.59, *p* = 0.03; Figure 6C). Thus, the combination of greater circumnutation frequency and larger circumnutation amplitude reduced friction at the cone‐soil interface. Furthermore, our calculations indicated that the reduction of the interfacial frictional force due to increased circumnutation intensity is more pronounced at low interfacial friction coefficients that typically occur at root‐soil interfaces (Figure 6D,E).

## 4 Discussion

Here, we combined measurements with customised penetrometers mimicking roots and X‐ray computed tomography imaging with a cavity expansion model to elucidate mechanisms underlying the effects of circumnutation on the mechanical resistance exerted on growing roots. We found that greater circumnutation intensity, i.e., the combination of high circumnutation frequency and amplitude, reduces interfacial friction, leading to lower mechanical resistance to soil penetration. These findings suggest that genotypic differences in root circumnutation amplitude (Taylor et al. 2021) and frequency (Inoue et al. 1999) can be leveraged to improve the exploration of hard soil.

The penetrometers used here as root analogues had a cone semi‐opening angle of 15° and a diameter‐to‐length ratio of around 0.1, which resembles the dimensions of root tips (Iijima, Barlow, and Bengough 2003; Colombi et al. 2017) and root growth zones (Bizet, Hummel, and Bogeat‐Triboulot 2015; Colombi et al. 2019, 2023; Quiros et al. 2022), respectively. Theoretically, root bending stiffness depends to the fourth power on root diameter and the logarithm of bending stiffness and diameter therefore scale linearly with a slope of four (Jin et al. 2013). A direct comparison of the mechanical behaviour of penetrometer probes and roots is difficult due to differences in material complexity between metal rods and complex biological tissues. Nevertheless, the bending stiffness and diameter of our penetrometer probes scaled well with data obtained from plant roots (slope = 4.40; Figure 2B; Dexter and Hewitt 1978). The difference in bending stiffness between stiffer steel and more flexible brass shafts (Figure 2A) resulted in circumnutation amplitudes varying from 28% to 32% of the cone base radius (Figure 3), which is within the range of circumnutation amplitudes of roots (Kim et al. 2016; Del Dottore et al. 2016; Martins et al. 2020; Taylor et al. 2021). Orbital movement of the sample at different velocities allowed us to simulate circumnutation frequencies of 1–10 oscillations per centimetre of penetration, which represents the variability in root circumnutation frequency of plants (Kim et al. 2016; Del Dottore et al. 2016; Martins et al. 2020; Taylor et al. 2021; Colombi et al. 2023). Hence, our experimental set‐up allowed simulating different root circumnutation intensities.

In contrast to previous studies conducted with granular (Tonazzini et al. 2012; Martins et al. 2020) or loosely packed substrate (Del Dottore et al. 2016, 2018), we measured mechanical resistance in field soil with a bulk density (*ρ_b_* = 1.4 g cm^−3^) and water content (*θ_g_* = 0.22 g g^−1^) representative for agricultural soil. Simulation models have shown that mechanical resistance decreases with circumnutation frequency (Chen and Martinez 2023). The findings obtained here with penetrometer probes that showed similar bending behaviour as plant roots (Figure 2B) corroborate these theoretical findings. In addition, our study highlights effects of circumnutation amplitude on the mechanical resistance to soil penetration. For probes with a stiffer steel shaft, which enabled a larger circumnutation amplitude than the more flexible brass shaft, circumnutation at one and five oscillations per centimetre of penetration decreased mechanical resistance by 10%. Such a reduction in mechanical resistance at one and five oscillations per centimetre of penetration did not occur for probes with a brass shaft (Figure 4). Similar effects of circumnutation amplitude on mechanical resistance have been obtained with root‐inspired robots penetrating sawdust (Del Dottore et al. 2016). Cell shortening in the root growth zone as observed in response to increased mechanical resistance (Croser, Bengough, and Pritchard 2000) can increase root stiffness (Liu et al. 2022) and therefore the potential circumnutation amplitude. Moreover, larger root circumnutation amplitudes upon greater mechanical resistance to soil penetration have been reported (Martins et al. 2020). Here we show that a larger circumnutation amplitude and a greater circumnutation frequency reduce mechanical resistance, indicating a link between circumnutation intensity and root growth rate in hard soil.

Soil structural properties obtained from X‐ray computed tomography scans at 12 µm resolution did not support the hypothesis that circumnutation promotes crack formation (Del Dottore et al. 2018) or alters soil deformation patterns around root tips (Vollsnes, Futsaether, and Bengough 2010). Mean pore diameter below the cone imprint was not affected by soil penetration, indicating that no visible cracks were formed below the cone (Supporting Information S1: Figure S3) and that circumnutation did not promote crack formation. However, such effects could occur in more brittle soils. We observed substantial impacts of soil penetration on soil density extending beyond the immediate vicinity of the cone imprint. The magnitude and relative extent (i.e., normalised to root radius) of these radial compression patterns (Figure 5B,C) was comparable to soil compression patterns occurring around plant roots (Dexter 1987; Bruand et al. 1996). However, neither these radial density profiles (Figure 5B,C) nor the size of the cone imprint (Figure 5A) differed among circumnutation frequencies or shaft materials, and thus circumnutation amplitudes. Hence, we did not find significant effects of circumnutation intensity on soil structure. This suggests that in the current study, cavity expansion forces were similar across circumnutation intensities and that changes in frictional force caused the effects of circumnutation on mechanical resistance. Similar to a penetrometer that rotates around its own axis, circumnutation changes the orientation of the frictional force vector, thereby reducing the frictional force (Bengough et al. 1991, 1997; McKenzie et al. 2013). These effects of penetrometer rotation on mechanical resistance are more pronounced at greater rotation frequencies (Bengough, Mullins, and Wilson 1997; Tang and Tao 2022), which corresponds to the relationship between increasing circumnutation frequency and decreasing mechanical resistance observed in our study (Figure 4).

Circumnutation and the resulting unidirectional radial force leads to an asymmetric distribution of the radial force acting on a penetrometer cone or a root tip penetrating soil (Figure 6A). The increase in root stiffness (Croser, Bengough, and Pritchard 2000; Liu et al. 2022) and root circumnutation amplitude (Martins et al. 2020) upon greater mechanical resistance indicate that plants actively promote an asymmetric distribution of the radial force around root tips when exposed to hard soil. In the current study, this asymmetry was determined by the bending stiffness of the penetrometer probe and the resulting circumnutation amplitude (Equations 11 and 12). Therefore, the stiffer steel shaft resulted in a more asymmetric distribution of the radial force around the cone than the more flexible brass shaft (Figure 6B). To quantify circumnutation intensity, we merged the degree of asymmetry and the circumnutation frequency into a single circumnutation intensity index. The negative relationship between circumnutation intensity and frictional force (Figure 6C) suggests that the combination of high circumnutation frequency and amplitude enables plants to reduce the mechanical resistance to soil penetration. Furthermore, we provide evidence that the relative reduction of the frictional force due to greater circumnutation intensity increases with lower interfacial friction coefficients (Figure 6D,E). Hence, effects of circumnutation on frictional forces are likely more pronounced for roots than for cone penetrometers due to lower friction coefficients occurring at root‐soil than at metal‐soil interfaces (Bengough and McKenzie 1997; McKenzie et al. 2013; Colombi et al. 2017). This indicates synergistic effects between lubrication of the rhizosphere through sloughing of root cap cells (Iijima, Griffiths, and Bengough 2000, 2003) and greater circumnutation intensity (Croser, Bengough, and Pritchard 2000; Martins et al. 2020; Liu et al. 2022; Chen and Martinez 2023) in hard soil.

## 5 Conclusions

Here, we provide mechanistic evidence suggesting that root circumnutation decreases friction at the root‐soil interface, which reduces the mechanical resistance to soil penetration. Mimicking different circumnutation behaviour with cone penetrometer analogues showed that these effects of circumnutation on mechanical resistance increase with circumnutation frequency and amplitude. Our study highlights the potential to leverage differences in root circumnutation behaviour and underlying root traits to adapt crops to hard soil. To further elucidate this potential, circumnutation effects must be tested across soil textures and densities. Performing such studies under different degrees of soil structural complexity, ranging from sieved to field structured soil will be key to establish robust linkages between soil mechanical conditions, root circumnutation and root foraging behaviour.

## Conflicts of Interest

The authors declare no conflicts of interest.

## Supporting information

Supporting information.

## Data Availability

The data that support the findings of this study are available from the corresponding author upon reasonable request.
