# Supplementary material for: Root Circumnutation Reduces Mechanical Resistance to Soil Penetration
Source: Plant Cell Environ. 2024 Oct 27;48(2):1608–20. doi: 10.1111/pce.15219 (PMC11695795; doi:10.1111/pce.15219)
Supplement: Supplementary file 1 — Supporting information. [file PCE-48-1608-s001.docx]

**Supporting information to:
Root circumnutation reduces mechanical resistance to soil penetration**

Frederic Leuther^1,2^, Daniel Iseskog^2^, Thomas Keller^2,3^, Mats Larsbo^2^, Bipin K. Pandey^4^, Tino Colombi^2,4,*^

^1^Chair of Soil Physics, University of Bayreuth, 95447 Bayreuth, Germany

^2^Department of Soil and Environment, Swedish University of Agricultural Sciences (SLU), P.O. Box 7014, 75007 Uppsala, Sweden

^3^Department of Agroecology and Environment, Agroscope, Reckenholzstrasse 191, 8046 Zürich, Switzerland

^4^School of Biosciences, University of Nottingham, Sutton Bonington, LE12 5RD, United Kingdom

^*^Corresponding author; email address: [tino.colombi@nottingham.ac.uk](mailto:tino.colombi@nottingham.ac.uk)

Supplemental Tables: 5
Supplemental Figures: 4

**SUPPLEMENTAL TABLES**

Supplemental Table S1: Basic soil properties of the central subsample and the surrounding bulk soil. For ρ_b_ and GWC, average values and (in brackets) standard deviations including all individual samples are shown (n = 45). Treatment mean values of ρ_b_ and GWC are shown in Supplemental Figure S1.

| **Soil property** | **Central subsample** | **Bulk soil** |
| --- | --- | --- |
| Clay/silt/sand [g g^-1^] | 0.15/0.64/0.21 | |
| SOM [g kg^-1^] | 41 | |
| ρ_b_ [g cm^-3^] | 1.40 (±0.012) | 1.40 (±0.004) |
| GWC [g g^-1^] | 0.22 (±0.005) | 0.22 (±0.005) |
| Abbreviations: SOM = soil organic matter; ρ_b_ = dry soil bulk density; GWC = gravimetric water content | | |

Supplemental Table S2: Camera and lens specifications and image acquisition parameters of RGB time-lapse imaging used to quantify the horizontal deflection of the penetrometer probe.

| **Parameter** | **Value** |
| --- | --- |
| Camera | Canon EOS M6, Canon, Tokyo, Japan |
| Lens | EF-M 28 mm f1/3 IS STM, Canon, Tokyo, Japan |
| Resolution | 4000 by 6000 pixels |
| Pixel edge length | 10.8 µm |
| Exposure time | 1/15 s |
| Aperture value | f/4 |
| Film speed | 100 ISO |

Supplemental Table S3: Acquisition and reconstruction parameters used for X-ray computed tomography imaging.

| **Parameter** | **Value** |
| --- | --- |
| Current | 130 μA |
| Voltage | 92 kV |
| Filter | 0.1 mm copper |
| Illumination time | 500 ms |
| Detector shift | on |
| Auto scan optimiser | on |
| Detector sensitivity | 1 |
| Averaged images | 2 |
| Skipped images | 1 |
| Acquired images per scan | 2000 |
| Voxel edge length | 12 μm |
| Beam hardening correction | 7 |
| Format exported images | .tiff (16 bit) |

Supplemental Table S4: Parameters used for processing of X-ray computed tomography images in FIJI ImageJ (Schindelin *et al.* 2012).

| **Parameter** | **Value** |
| --- | --- |
| Gray value calibration | Pores = 5000, cylinder =25000 grey values |
| Non-local means filter | Sigma of noise = 12 |
| Unsharp mask | Radius = 1, mask=0.60 |
| Global threshold | 70 grey values |
| Median 3D | Radius = 10 |

Supplemental Table S5: Comparison of measured penetration force in vertical direction (F_z_) and actual axial penetration force (F_a_) after correction for horizontal deflection of the probe caused by circumnutation for different shaft materials and circumnutation frequencies (CF). Probe deflection and F_a_ were calculated using the average difference between maximum and minimum shaft angle (∆α) as shown in Eq. 1 and 2, respectively and the relative error (ε_rel_) between F_z_ and F_a_ was calculated as shown in Eq. 3. Mean values of five replicates are shown (n = 5).

| **Material** | **CF [# cm^-1^]** | **∆α [°]** | **Deflection [mm]** | **F_z_ [mN]** | **F_a_ [mN]** | **ε_rel_ [%]** |
| --- | --- | --- | --- | --- | --- | --- |
| Steel | 0 | 0 | 0 | 15405.21 | 15405.21 | 0 |
|  | 1 | 0.50 | 0.19 | 13794.10 | 13794.23 | <0.001 |
|  | 5 | 0.59 | 0.23 | 13967.94 | 13968.13 | 0.001 |
|  | 10 | 0.57 | 0.22 | 13223.63 | 13223.79 | 0.001 |
| Brass | 0 | 0 | 0 | 15424.36 | 15424.36 | 0 |
|  | 1 | 0.75 | 0.30 | 15545.75 | 15546.08 | 0.002 |
|  | 5 | 0.78 | 0.31 | 15402.83 | 15403.18 | 0.002 |
|  | 10 | 0.73 | 0.29 | 13334.32 | 13334.59 | 0.002 |

**SUPPLEMENTAL FIGURES**


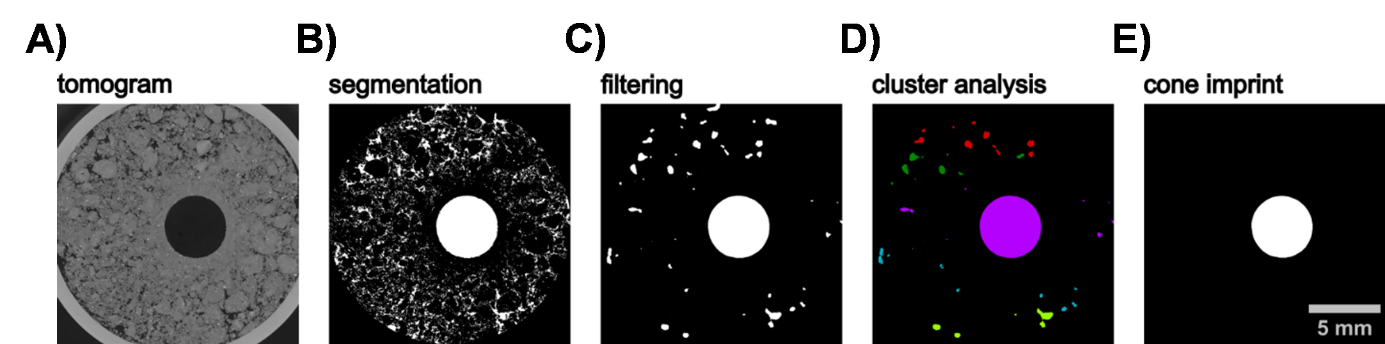


Supplemental Figure S1: Step-by-step illustration of image processing procedure to isolate cone imprint in X-Ray computed tomography scans at a resolution of 12 µm. A) Tomogram of a typical cross-section, B) binary image of the cross-section after segmentation, C) binary image after filtering to remove pores below 120 µm diameter, D) result of cluster analysis to identify individual pore clusters and E) final isolation of the largest pore cluster represented by the cone imprint. Scale bar displayed in E) applies to all panels. Detailed image analysis parameters are provided in Supplemental Table S4.


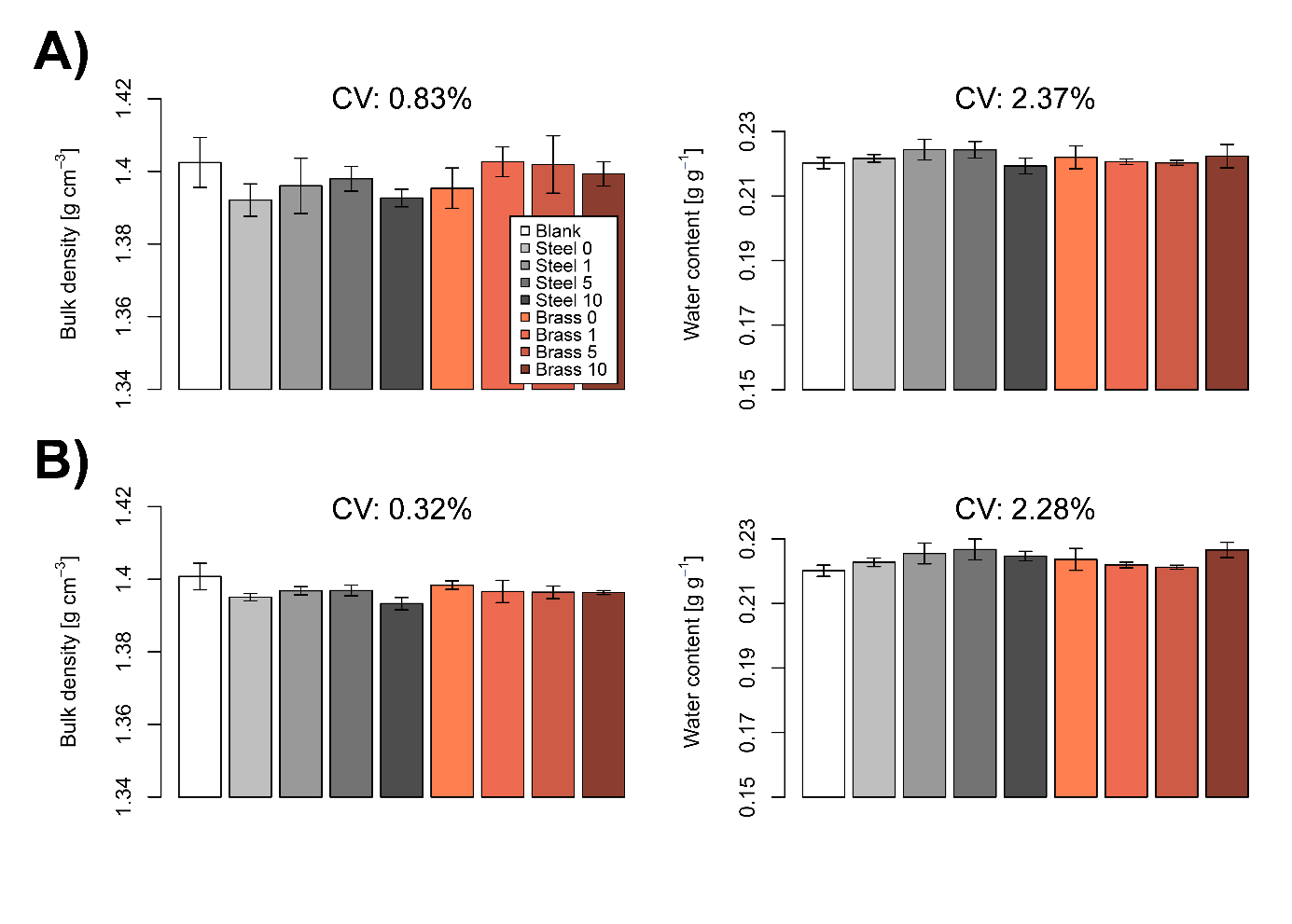


Supplemental Figure S2: Dry soil bulk density and gravimetric water content of A) central subsample and B) surrounding bulk soil of samples penetrated with probes with a steel (grey bars) or brass (brown bars) shaft or samples that were not subjected to penetrometer tests (white bars). Numbers denote circumnutation frequencies in oscillations per centimetre of penetration. CV values represent coefficient of variation across all samples and error bars denote standard error (n = 5).

Supplemental Figure S3: Soil structural features obtained from X-ray computed tomography scans at a resolution of 12 µm. A) visible soil porosity (ε) and B) mean pore diameter. Top and middle panels depict properties obtained from the segment of the sample around and below the cone imprint, respectively, and bottom panels show average values of top and middle panels. Blank denotes samples not subjected to penetrometer tests. P-values were obtained from analysis of co-variance models testing effects of circumnutation frequency (CF), shaft material (M), and their interaction (CF:M), excluding blanks (n = 5).


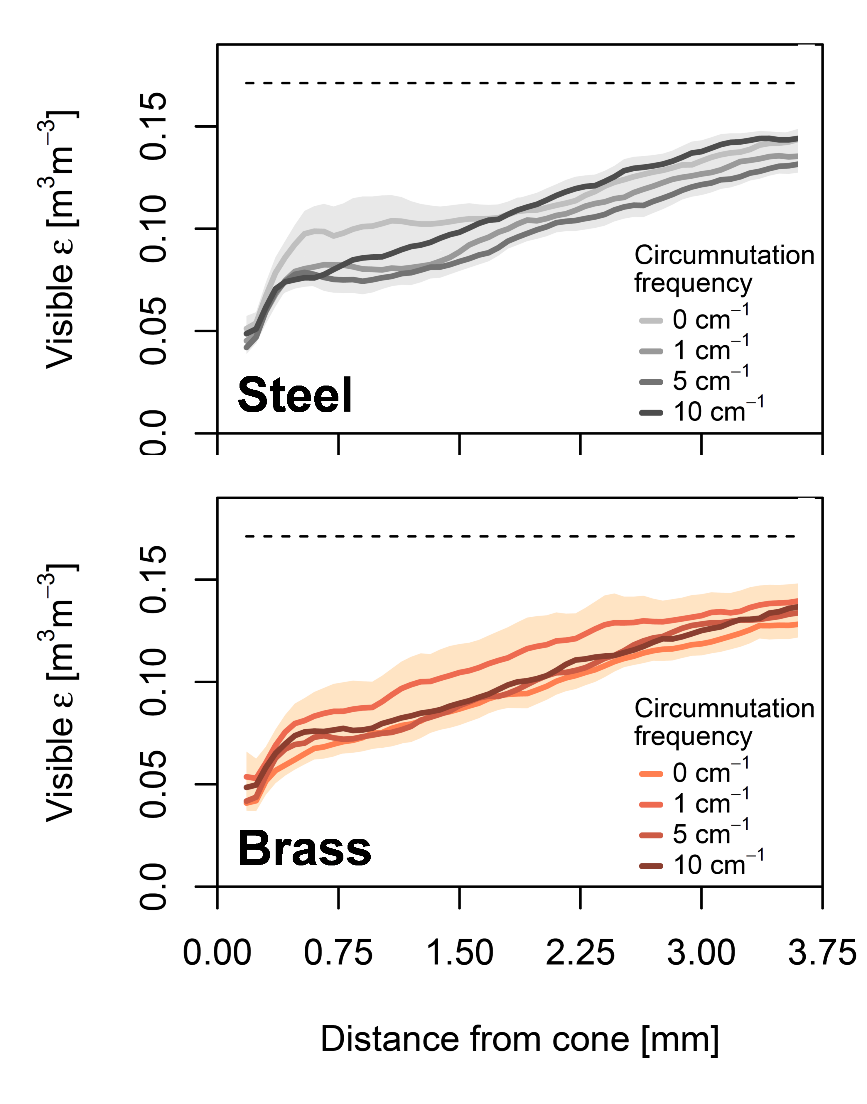


Supplemental Figure S4: Visible soil porosity obtained from X-ray computed tomography scans (12 µm resolution) around the cone imprint as a function of the distance from the surface of the cone imprint. Dashed line indicates average visible porosity measured in samples not subjected to penetrometer tests and shading denotes standard error (n = 5).

**CITED LITERATURE**

Schindelin J., Arganda-Carreras I., Frise E., Kaynig V., Longair M., Pietzsch T., … Cardona A. (2012) Fiji: An open-source platform for biological-image analysis. *Nature Methods* **9**, 676–682.
